# First Outbreak of Dengue Hemorrhagic Fever, Bangladesh

**DOI:** 10.3201/eid0807.010398

**Published:** 2002-07

**Authors:** Mahbubur Rahman, Khalilur Rahman, A. K. Siddque, Shereen Shoma, A. H. M. Kamal, K. S. Ali, Ananda Nisaluk, Robert F. Breiman

**Affiliations:** * ICDDR,B: Centre for Health and Population Research, Dhaka, Bangladesh; † Holy Family Hospital, Dhaka, Bangladesh; ‡ The Armed Forces Research Institute of Medical Sciences, Bangkok, Thailand

**Keywords:** Dengue, dengue hemorrhagic fever, dengue shock syndrome, dengue outbreak, Bangladesh, primary dengue infection, secondary dengue infection

## Abstract

During the first countrywide outbreak of dengue hemorrhagic fever in Bangladesh, we conducted surveillance for dengue at a hospital in Dhaka. Of 176 patients, primarily adults, found positive for dengue, 60.2% had dengue fever, 39.2% dengue hemorrhagic fever, and 0.6% dengue shock syndrome. The Dengue virus 3 serotype was detected in eight patients.

Dengue fever (DF) and dengue hemorrhagic fever (DHF) are caused by four antigenically distinct but related dengue virus (official name: *Dengue virus* [DENV]) serotypes transmitted primarily by *Aedes aegypti* (yellow fever mosquito). DHF, the severe form of the disease, is endemic and frequently intensifies into epidemics in Southeast Asia, resulting in frequent hospitalizations and deaths (1,2). Recently, dengue has emerged as a substantial global health problem with increased incidence in new countries and tropical areas (3,4). DF was documented in Bangladesh from the mid-1960s to the mid-1990s, but an outbreak of DHF has not been previously reported (5,6). During late June 2000, a 28-year-old patient was admitted to a hospital in Dhaka, Bangladesh, with hemorrhagic fever, ascites, pleural effusion, and thrombocytopenia. An enzyme-linked immunosorbent assay (ELISA) for anti-dengue antibodies confirmed the case as DHF (1). That summer, an outbreak of DF (>5,000 hospitalized cases reported) and DHF occurred in Dhaka and other major cities of Bangladesh (7).

## The Study

We began surveillance for dengue among patients at a hospital in Dhaka during July 1–October 31, 2000. Clinical details of each patient were recorded on a standardized form; when indicated, chest radiographs and abdominal ultrasounds were done, in addition to hemoglobin, hematocrit, and total blood and platelet counts. Sera from all patients were tested by ELISA for anti-dengue and anti-Japanese encephalitis viral immunoglobulin (Ig)M and IgG (8). Paired sera were tested when available. Sera from 30 patients with fever of <6 days’ duration were also tested for serotype-specific dengue viral RNA by reverse transcription-polymerase chain reaction (RT-PCR) (9). Samples having >40 units of IgM or IgG antibodies were considered positive for dengue infection. A ration of IgM and IgG <1.8 defined a secondary infection and >1.8 a primary infection (8). DF, DHF, and dengue shock syndrome (DSS) were defined according to World Health Organization (WHO) criteria (1) and confirmed by positive IgM or IgG ELISA or RT-PCR. Statistical analysis was done by chi-square test.

Of 336 suspected dengue patients, sera were available for 240 patients; 176 (73.3%) had confirmed dengue infection (168 by ELISA; 2 by RT-PCR; and 6 by both tests). RT-PCR detected dengue virus 3 serotype (DENV-3) in 8 (26.6%) of 30 patients tested. Both acute- and convalescent-phase sera were positive in 18 of 30 paired specimens available, and seroconversion occurred in 2 of the remaining 12 sera. All sera were negative for antibodies to *Japanese encephalitis virus*. Thirty-one (18%) cases were in children (<18 years of age). The highest proportion of cases occurred in persons 18–33 years of age (Figure). DF occurred most commonly (60.2%), followed by DHF (39.2%), and DSS (0.6%). Most (80%) patients reported to the hospital after 5 days of fever. Spontaneous bleeding occurred in 91% of patients with DHF, compared with 25% patients with DF (p<0.01). Frequent clinical features were fever (100%), headache (91%), myalgia/arthralgia (85%), vomiting (64%), macular rash (55%), bleeding (46%) (including melena [20%] and bleeding gums [11.6%]), thrombocytopenia (<100,000/mL, 56.7%), pleural effusion (12%), ascites (9%), and hepatomegaly (7.5%). Secondary infection was detected in 71% of 174 ELISA-positive cases, more commonly in patients with DHF than with DF (relative risk 2.14, p=0.002) (Table).

**Figure:**
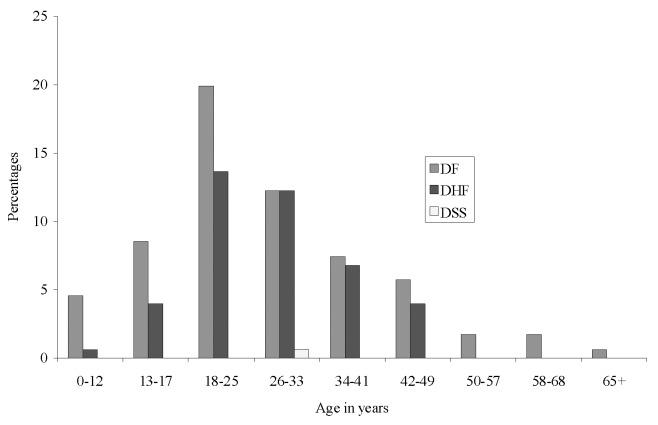
Age distribution of dengue cases, Bangladesh, 2000

**Table:** Distribution of serologically positive dengue cases by type of infections in adults and children, Bangladesh, 2000

Type of infection, by age group^a^	No. (%) of dengue cases, by category			
	Dengue fever (n=104)	Dengue hemorrhagic fever (n=69)	Dengue shock syndrome (n=1)	Total cases (n=174)^b^
Primary Adults	39 (37.5) 26 (25.0)	11 (15.9) 10 (14.5)	0 (0) 0 (0)	50 (28.7) 36 (20.7)
Children	13 (12.5)	1 (1.4)	0 (0)	14 (8.0)
Secondary Adults	65 (62.5) 55 (52.9)	58 (84.1) 51 (73.9)	1 (100) 1 (100)	124 (71.3) 107 (61.5)
Children	10 (9.6)	7 (10.1)	0 (0)	17 (9.8)

^a^Adults >18 yrs of age; children <18 yrs of age. ^b^Two cases that were negative by enzyme-linked immunosorbent assay and positive by reverse transcription-polymerase chain reaction were not included.

All but two adult patients recovered (case-fatality rate 1.14%). One DF patient died with severe hematemesis and melena; another DSS patient died within 2 hours of hospitalization.

## Conclusions

Dengue causes more illness and death than any other arboviral infection in the world (4). This first outbreak highlights the geographic expansion of DHF in Bangladesh, where classic DF caused by multiple serotypes had been previously reported (5,6). The DHF outbreak started in late June 2000, peaked in September (during the rainy season), and subsided in the dry winter season in December 2000. While dengue affected all age groups, adults predominated in this hospital-monitored study. In Singapore, India, Malaysia, and Brazil, where dengue has been epidemic for several years, the mean age of dengue infection is increasing and adults are frequently infected, indicating an epidemiologic change in dengue infection in those locations (1,10).

The precise magnitude of this countrywide outbreak is unknown; 5,575 hospitalized dengue cases were reported to the Ministry of Health in Bangladesh, with a case-fatality rate of 1.61% through mid-November 2000 (6). Most patients had DF, 25% with bleeding manifestations (a severe form of the illness) (1,4). WHO classification of dengue diseases is often not feasible in many countries because of lack of trained health professionals, adequate laboratories, and radiologic support. The facilities to detect DHF by using hematocrit (capillary method) and plasma leakage signs (chest radiograph or ultrasound) are not readily available in many tropical countries. Successful treatment of dengue depends on symptom recognition and careful fluid management (1). Thus, a simple classification scheme of dengue diseases based on symptoms and signs is needed to improve case management and reduce deaths.

DHF is believed to occur as a result of antibody-dependent enhancement of heterotypic-secondary dengue infections (1,4,11). Our findings support the role of sequential infection in the development of DHF. However, occurrence of DHF in some patients with primary infection suggests additional host and virologic factors.

In our study, DSS was a rare event, resulting in a lower case-fatality rate for dengue than reported elsewhere (7), likely representing hospitalization of less severe cases. We have insufficient data to comment on the virulence of the outbreak strain. High numbers of *Ae. aegypti* were identified throughout the city (Y. Wagatsuma, ICDDR,B; pers. comm.). Insecticide spraying, public health education (including community source reduction), and perhaps most importantly, the onset of the dry winter season may have contributed to ending this dengue outbreak in 2000.

Therapeutic strategies proposed by WHO have been widely circulated by the Bangladesh government, and physicians continue to gain experience in proper management of dengue. Ongoing surveillance, vector surveys, and epidemiologic studies to identify risk factors will provide key information for controlling dengue. Ultimately, a safe and effective vaccine will be needed to address this emerging problem in Bangladesh and elsewhere.
